# Predicting Cognitive Decline Using Combined Plasma and Imaging Biomarkers: Evidence from a Singapore Memory Clinic Cohort

**DOI:** 10.1002/alz70856_100175

**Published:** 2025-12-24

**Authors:** Yuan Cai, Joyce R Chong, Mitchell Kim Peng Lai, Ho Ko, Vincent C.T. Mok, Christopher Li‐Hsian Chen

**Affiliations:** ^1^ Division of Neurology, Department of Medicine and Therapeutics, Faculty of Medicine, The Chinese University of Hong Kong, Prince of Wales Hospital, Hong Kong SAR, Hong Kong; ^2^ Lui Che Woo Institute of Innovative MedicineLi Ka Shing Institute of Health Science, The Chinese University of Hong Kong, Prince of Wales Hospital, Hong Kong SAR, Hong Kong; ^3^ The Chinese University of Hong Kong, Hong Kong, Hong Kong; ^4^ Memory, Ageing, and Cognition Centre (MACC), Department of Pharmacology, Yong Loo Lin School of Medicine, National University of Singapore, Singapore, Singapore; ^5^ Memory Aging and Cognition Center, National University Health System, Singapore, Singapore; ^6^ Yong Loo Lin School of Medicine, National University of Singapore, Singapore, Singapore; ^7^ Gerald Choa Neuroscience Institute, The Chinese University of Hong Kong, Hong Kong SAR, Hong Kong; ^8^ Lau Tat‐Chuen Research Centre of Brain Degenerative Diseases in Chinese, Faculty of Medicine, The Chinese University of Hong Kong, Hong Kong SAR, Hong Kong; ^9^ Li Ka Shing Institute of Health Sciences, Faculty of Medicine, The Chinese University of Hong Kong, Hong Kong SAR, Hong Kong; ^10^ Division of Neurology, Department of Medicine and Therapeutics, The Chinese University of Hong Kong, Hong Kong SAR, Hong Kong; ^11^ Margaret K. L. Cheung Research Centre for Management of Parkinsonism, Faculty of Medicine, The Chinese University of Hong Kong, Hong Kong SAR, Hong Kong; ^12^ Memory Ageing and Cognition Centre, National University Health System, Singapore, NA, Singapore; ^13^ Department of Pharmacology, Clinical Research Centre, National University Health System, National University of Singapore, Singapore, Singapore

## Abstract

**Background:**

The AD Resemblance Atrophy Index (AD‐RAI) has demonstrated superior performance compared to other neurodegeneration biomarkers in diagnosis and prognosis of Alzheimer's Disease (AD). Based on the 2024 NIA‐AA criteria, in addition to Amyloid (A), Tau (T), and Neurodegeneration (N, NfL, atrophy) biomarkers, Inflammation (I, GFAP) and Vascular (V, white matter hyperintensity [WMH]) biomarkers are recognized as co‐pathology biomarkers contributing to AD progression. These biomarkers have previously been reported to be associated with cognitive decline, however, there is currently a lack of studies exploring the performance of a combination of biomarkers in predicting early cognitive decline.

**Method:**

We recruited 314 non‐demented participants from a Singapore memory clinic who had either no cognitive impairment or mild cognitive impairment and were followed up for 2 years. Plasma biomarkers (GFAP, NfL, *p*‐tau181, Aβ42) and MRI measures (AD‐RAI, hippocampal volume, WMH) were collected at baseline. Cognitive decline was defined as a one‐point increase in the CDR‐SB score within 2 years.

**Result:**

Of the 314 participants, 77 (24.5%) showed cognitive decline within 2 years. Baseline CDR‐SB, AD‐RAI, plasma GFAP, APOE4 carrier status, and hippocampal volume were independently associated with cognitive decline after adjustments for age, gender, and education. Among the individual biomarkers analyzed using ROC, plasma GFAP demonstrated the best performance in predicting cognitive decline with an AUC of 0.73 (sensitivity: 85.7%; specificity: 51.9%). AD‐RAI out‐performed plasma NfL and all chronic brain change biomarkers, achieving an AUC of 0.72 (sensitivity: 74.0%; specificity: 63.7%). A parsimonious model, which included demographic factors, CDR‐SB, plasma biomarkers (GFAP, Aβ42), AD‐RAI, and WMH, achieved an optimal balance between model complexity and performance (AUC: 0.83; sensitivity: 85.7%; specificity: 66.2%).

**Conclusion:**

Incorporating demographic factors, CDR‐SB, plasma biomarkers (GFAP, Aβ42), AD‐RAI, and WMH demonstrated good predictive performance for cognitive decline. Combining co‐pathology biomarkers with AD‐RAI and plasma based Aβ biomarkers enhanced model performance, highlighting the potential of multi‐biomarker approaches in predicting cognitive decline. Future studies with larger, multi‐ethnic cohorts are needed to validate these findings and assess their generalizability

